# Friend or foe? Glial–vascular interactions in health and neurodegenerative disease

**DOI:** 10.1016/j.pharmr.2026.100143

**Published:** 2026-05-12

**Authors:** Conor McQuaid, Michael Sewell, Axel Montagne

**Affiliations:** 1 UK Dementia Research Institute at the University of Edinburgh, Edinburgh, United Kingdom; 2 British Heart Foundation – UK Dementia Research Institute Centre for Vascular Dementia Research at the University of Edinburgh, Edinburgh, United Kingdom; 3 Institute for Neuroscience and Cardiovascular Research (INCR), The University of Edinburgh, Edinburgh, United Kingdom

## Abstract

Dysfunction of glial and vascular cells is increasingly recognized as a central feature of neurodegenerative diseases. Growing evidence points to disruptions in glial-vascular interactions, which are critical for maintaining the functions of the neurogliovascular unit throughout the lifespan, as key contributors to disease initiation and progression. However, the mechanisms governing this complex intercellular crosstalk and its potential role in disease pathogenesis remain incompletely understood. In this review, we summarize the current understanding of glial–vascular communication across health and disease, with a particular focus on Alzheimer disease, stroke, cerebral small vessel disease, Parkinson disease, Huntington disease, and multiple sclerosis. We highlight emerging cellular and molecular interactions of interest, outline major gaps in our understanding, and discuss innovative tools, including transcriptomics, which are reshaping the study of neurogliovascular dynamics. A central unresolved question is whether glial and/or vascular dysfunction represents the primary initiating event across neurodegenerative diseases, or whether these processes emerge in parallel through shared upstream drivers. Unraveling these interactions may ultimately reveal novel therapeutic opportunities for a broad range of neurodegenerative conditions.

**Significance Statement:**

Neurogliovascular unit interactions are fundamental to brain homeostasis, yet the molecular basis of this crosstalk and its disruption in neurodegeneration remains poorly understood. This review provides the first comprehensive synthesis of molecular mechanisms governing the Neurogliovascular unit interface across physiological and pathological conditions, integrating evidence from related disorders. By consolidating key signaling pathways, disease-associated alterations, and emerging experimental approaches, this review offers a unifying framework to guide biomarker development and therapeutic targeting.

## I Introduction

**Fig. 1:**
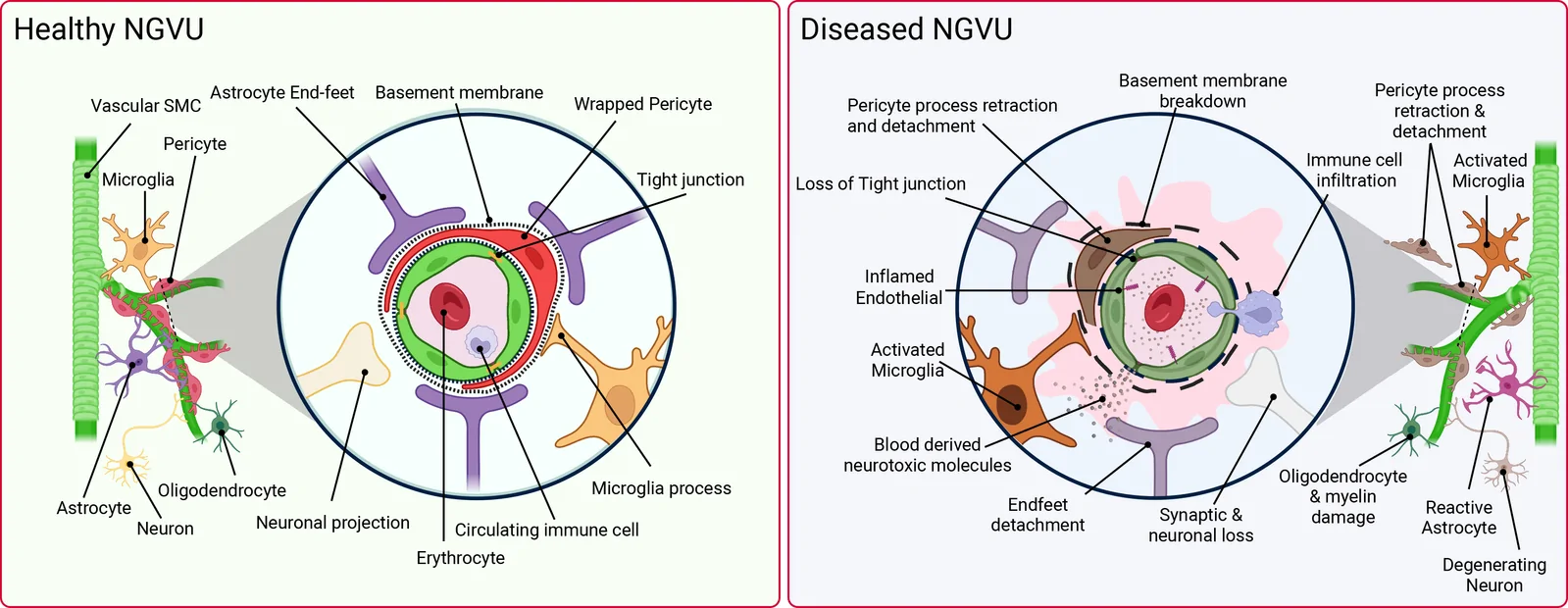
Overview of the healthy and diseased NGVU. (Left panel) The healthy NGVU comprises neurons, microglia, astrocytes, oligodendrocytes, pericytes, VSMCs, and ECs, which together maintain BBB integrity, CBF, and neurovascular coupling. Key structural features include intact TJs, astrocyte endfeet ensheathing the vasculature, and pericyte coverage of the basement membrane. (Right panel) In disease, coordinated dysfunction of the NGVU components leads to pericyte process retraction and detachment, basement membrane degradation, loss of TJs, endothelial activation and inflammation, astrocyte endfoot detachment, microglial activation, and oligodendrocyte and myelin damage. These changes permit the entry of blood-derived neurotoxic molecules into the parenchyma, promote peripheral immune cell infiltration, and ultimately contribute to synaptic dysfunction and neuronal loss. Created with BioRender.com.

The neurogliovascular unit (NGVU) is a highly complex, dynamic, and multicellular network within the central nervous system (CNS) that is essential for maintaining brain health and function. By regulating cerebral blood flow (CBF) and preserving the integrity of the blood-brain barrier (BBB), the NGVU ensures the brain’s metabolic needs are met, a process known as neurovascular coupling. In addition, the NGVU is increasingly recognized for its role in modulating immune responses within the CNS. These diverse functions rely on tight coordination and bidirectional crosstalk between its key cellular components: neurons, glial cells (microglia, astrocytes, and oligodendrocytes), and vascular cells (endothelial and mural cells) (Fig. 1).1., 2., 3.

In recent years, the NGVU has emerged as a central player in the pathophysiology of several CNS disorders, including Alzheimer disease (AD),^4^ stroke,^5^^,^^6^ cerebral small vessel disease (cSVD),^7^ Parkinson disease (PD),^8^^,^^9^ Huntington disease (HD),^10^ and multiple sclerosis (MS).^11^ Emerging evidence suggests that NGVU dysfunction, including BBB alterations, reduced CBF, and impaired angiogenesis, often precedes and exacerbates neuronal deficits and cognitive decline in these diseases.^4^ When the BBB is compromised, blood-derived neurotoxic molecules, including fibrinogen, thrombin, and albumin, can enter the brain parenchyma, triggering glial activation, and exacerbating neuronal damage. As such, the interaction between the cells of the NGVU represents both a potential biomarker and a promising therapeutic target in neurodegeneration.

Despite growing interest, our understanding of NGVU dysfunction remains limited. One area that has received increased attention is the disruption of communication between glial and vascular cells within the NGVU. Alterations to glial and vascular cell profiles have been extensively documented in neurodegenerative diseases (NDDs),^12^^,^^13^ and such changes likely impair their communication, promoting NGVU breakdown, and exacerbating disease pathology. Ultimately, these disruptions to glial–vascular communication compromise the metabolic and structural support that neurons depend upon, contributing to synaptic dysfunction and neuronal loss, the defining clinical outcomes of NDD.

In this review, we first describe the roles of the individual glial and vascular components within the NGVU and how they interact with one another to maintain CNS homeostasis. We then discuss findings implicating glial-vascular interactions in various NDDs and associated pathologies. Finally, we outline knowledge gaps and future directions for advancing our understanding of NGVU dysfunction, highlighting the promise of emerging cutting-edge technologies, such as omics approaches, to decode this complexity.

## II Glia and vascular components of the neurogliovascular unit

### A Vascular cells

#### 1 Endothelial cells

Endothelial cells (ECs) are the primary vascular components of the NGVU, forming the inner lining of blood vessels and playing a central role in maintaining the BBB. In the CNS, ECs are highly specialized compared with those in other tissues. They exhibit features such as reduced pinocytosis and transcytosis, elevated expression of tight junction (TJ) proteins (eg, claudin-5, occludin, and zonula occludens-1 [ZO-1]), and restricted transport of substances larger than ∼400 Da.^14^ These adaptations enhance BBB integrity and enable ECs to tightly regulate exchange between the blood and the brain parenchyma, thereby supporting CNS homeostasis.^15^^,^^16^

Advances in single-cell and single-nucleus RNA sequencing (snRNAseq) have revealed substantial regional heterogeneity in ECs, particularly along the arteriovenous axis and across CNS regions, with distinct transcriptional profiles.17., 18., 19., 20., 21. For example, arterial ECs are enriched in angiogenesis-related genes, capillary ECs exhibit high expression of BBB-associated transporters, such as major facilitator superfamily domain-containing protein 2 (MFSD2A) and SLC7A5, and venous ECs show increased expression of genes linked to inflammatory signaling,^19^^,^^20^^,^^22^ suggesting additional roles for ECs in processes such as CNS inflammation.

Beyond zonal differences, ECs also display regional heterogeneity across brain areas, likely reflecting the unique functional demands of each region.^17^^,^^23^^,^^24^ In the entorhinal cortex, a region vulnerable to early AD pathology, ECs upregulate cytokines such as C-X-C motif chemokine ligand 12, which can attract microglia.^25^ In the prefrontal cortex, ECs show enriched expression of genes associated with microtubule organization (eg, TUBB2B), suggesting a role in structural plasticity.^26^ Meanwhile, ECs in the visual cortex exhibit elevated expression of antiviral response genes (eg, IFITM3) and angiogenesis-related pathways, consistent with this region’s high metabolic demands for visual processing.^24^^,^^27^ Such findings highlight the remarkable functional plasticity of ECs in supporting the diverse physiological needs across brain regions.

#### 2 Pericytes

Pericytes are mural cells embedded within the basement membrane of brain microvessels, strategically positioned at the interface between the blood and brain parenchyma.^28^ This unique location allows them to receive and integrate signals from neighboring cells, positioning them as central regulators of neurovascular function.^14^^,^^29^ However, compared with ECs, the nature of these interactions and the roles of pericytes are far less well characterized.

A primary function of pericytes is to provide structural support and regulate blood vessel diameter and flow.^29^^,^^30^ Through their contractile properties, they help modulate capillary hemodynamic responses and contribute to the fine-tuning of CBF.^29^^,^^31^^,^^32^ This regulation is crucial for ensuring proper brain perfusion and meeting the dynamic metabolic demands of neural tissue.^33^ Pericytes also play an essential role in maintaining BBB integrity from early developmental stages,^34^ partly by releasing signaling molecules that regulate permeability and support vascular development and maturation.^32^

Similar to their EC counterparts, pericytes contribute to neuroinflammation and microglial homeostasis by modulating the secretion of inflammatory cytokines.^35^^,^^36^ Recent evidence also suggests that pericytes can influence neuronal activity through the secretion of factors such as insulin-like growth factor 2.^37^ In certain contexts, pericytes may even differentiate into other CNS cell types, including microglia-like cells.^38^^,^^39^ These findings suggest that the role of pericytes extends well beyond vascular support and encompasses broader contributions to CNS signaling and homeostasis.^40^

#### 3 Smooth muscle cells

Vascular smooth muscle cells (VSMCs) are mural cells primarily located in the walls of arterioles and small arteries. Highly contractile, they work alongside pericytes to regulate blood vessel diameter and CBF in response to neurotransmitters, hormones, and local metabolic cues.^41^ A key feature of VSMCs is their phenotypic plasticity, the ability to transition between contractile and synthetic states, which enables adaptation to diverse physiological demands.^40^^,^^41^

Although VSMCs are not direct components of the BBB, they help maintain its integrity by supporting the function of ECs and pericytes.^14^ VSMCs are vital for neurovascular coupling, collaborating with other NGVU components to adjust CBF in response to neuronal activity.^42^ In addition, they are instrumental in the development and maturation of the cerebral vasculature, contributing to long-term vascular stability and resilience.^29^

### B Glial cells

#### 1 Microglia

Microglia are the resident macrophages of the CNS and brain parenchyma. They represent a functionally heterogeneous population involved in both immune and nonimmune processes throughout life.^43^^,^^44^ Their roles include modulating BBB integrity, regulating CBF, and participating in neurovascular coupling, functions that are believed to depend largely on purinergic signaling via microglial-expressed receptors such as P2Y12, which acts in conjunction with pannexin-1 hemichannels to modulate vascular tone.45., 46., 47. The importance of microglia in vascular regulation is underscored by the presence of vessel-associated microglia, which are in direct contact with blood vessels and are observed as early as during development.^46^^,^48., 49., 50. Vessel-associated microglia have been implicated in key processes such as phagocytosis and angiogenesis, particularly during early CNS development,^3^ emphasizing their essential importance to vascular integrity across the lifespan. Although these pathways are primarily involved in neuron–microglia communication, they are briefly noted here as they modulate microglial behavior at the vessel wall and can influence CBF and NGVU function, making them relevant to glial-vascular regulation microglia also maintain homeostatic communication with neurons via the CX3CL1-CX3CR1 (C-X3-C motif chemokine ligand 1 [fractalkine]–C-X3-C motif chemokine receptor 1 [fractalkine receptor]) signaling axis, whereby neuronal CX3CL1 signals to microglial CX3CR1 to promote a surveillance phenotype and limit excessive microglial activation.^51^ Beyond neuron-microglia communication, CX3CR1 signaling also contributes directly to glial-vascular regulation, with microglial CX3CR1 implicated in cerebrovascular blood flow adaptation during hypoperfusion and in vasoregulation at the vessel wall, with CX3CR1 deficiency associated with impaired vascular integrity.

#### 2 Astrocytes

Astrocytes are glial cells that play critical roles in maintaining the cellular, molecular, and metabolic homeostasis within the NGVU.^52^^,^^53^ Astrocytic endfeet are thought to contribute to waste clearance, although the exact mechanisms remain incompletely understood.^52^ In disease states, astrocyte endfeet undergo structural remodeling, including retraction from the vascular surface and disruption of aquaporin-4 polarization, which further compromises astrocyte-endothelial communication and glymphatic function.

#### 3 Oligodendrocytes

Oligodendrocytes are specialized glial cells in the CNS best known for their role in myelination and axonal support. However, they are increasingly recognized as active participants in vascular regulation. Recent research has shown that oligodendrocyte precursor cells (OPCs) interact closely with NGVU components and that mature oligodendrocytes can also make direct contact with blood vessels.^54^^,^^55^ Oligodendrocytes contribute to BBB integrity by regulating the expression and maintenance of TJ proteins and influencing the expression of transporters and specialized enzyme systems that constitute the transport and metabolic barriers of the BBB.^56^ Notably, oligodendrocyte-derived laminin-*γ*1 has been identified as a key factor required for BBB maintenance.^57^

Both oligodendrocytes and their precursors have been implicated in angiogenesis and vascular remodeling. OPCs can stimulate angiogenesis during development through hypoxia-inducible factor-dependent secretion of Wnt proteins and vascular endothelial growth factor (VEGF), presumably to adapt local blood supply to the metabolic needs associated with OPC differentiation.^58^^,^^59^

## III Glial-vascular interactions supporting neurogliovascular unit function

### A Blood-brain barrier maintenance

The maintenance of BBB integrity relies heavily on the intricate interactions between the various cell types of the NGVU. Although the importance of intervascular cell communication in BBB maintenance is well established,^28^^,^^33^^,^^34^ more recent research has highlighted critical roles for glial-vascular crosstalk, particularly involving astrocytes. Astrocytes further support BBB integrity by regulating the expression and maintenance of TJ proteins, including occludin, claudin-5, and ZO-1, all of which are essential for BBB function and for limiting paracellular permeability.^60^ Astrocytes also control the expression and polarized localization of transporters and specialized enzymatic systems that comprise the transport and metabolic barriers of the BBB.^61^^,^^62^ They secrete growth factors that contribute to the maintenance and repair of the BBB.^53^ For instance, astrocyte-secreted apolipoprotein E interacts with low-density lipoprotein receptor-related protein 1 (LRP1) on pericytes to suppress the cyclophilin A-nuclear factor *κ*B-matrix metalloproteinase-9 pathway, thereby preserving BBB integrity.^53^^,^^63^ Astrocyte-derived laminin is also thought to contribute to this process by modulating pericyte differentiation, further supporting BBB function.^64^ Additionally, astrocyte–EC associations in regulating BBB integrity have recently been demonstrated in vivo, with studies showing that the astrocytic solute carrier family 4 member 4–C-C motif chemokine ligand 2 (Slc4a4-CCL2) and endothelial C-C chemokine receptor type 2 axis specifically modulates barrier stability.^65^ Moreover, astrocytes are also believed to release factors that influence the expression of TJ proteins in ECs.^66^

The primary signaling pathways in EC–pericyte interactions under physiological conditions include neurogenic locus notch homolog protein 3 (NOTCH3)-Jagged1, platelet-derived growth factor receptor *β* (PDGFR*β*)-PDGF-BB, N-cadherin, Tie2-angiopoietin, transforming growth factor *β* (TGF-*β*)-TGFBR2, and MFSD2A.^67^^,^^68^

NOTCH3 is expressed in brain pericytes and VSMCs and plays a crucial role in maintaining cerebrovascular integrity. NOTCH3 signaling is activated through direct cell-to-cell contact, promoting pericyte population expansion, likely via positive regulation of PDGFR*β*.^7^^,^^28^^,^^67^^,^^69^^,^^70^ Mutations in NOTCH3 cause cerebral autosomal dominant arteriopathy with subcortical infarcts and leukoencephalopathy, the most common hereditary cSVD, which affects both VSMCs and pericytes.^71^ PDGF-BB/PDGFR*β* signaling is critical for pericyte recruitment during brain vascular development, and endothelial expression and secretion of PDGF-BB are required to maintain pericyte coverage and normal BBB function. Loss of PDGF-BB leads to reduced pericyte coverage and BBB breakdown.^28^^,^^67^ Angiopoietin-1 secreted by pericytes, binds to the Tie2 receptor on ECs, promoting BBB integrity by stabilizing endothelial junctions and TJ protein expression (occludin and ZO-1) while reducing vascular permeability.^72^ Conversely, angiopoietin-2, secreted by ECs, can inhibit angiopoietin-1-Tie2 binding, enabling ECs to regulate receptor activation. Disrupted angiopoietin-Tie2 signaling has been observed in stroke, diabetes, and other vascular diseases.^68^^,^^72^ N-cadherin forms adherens junctions between ECs and pericytes and is critical for BBB integrity. N-cadherin mediates adhesion, recognition, and signaling between these cells through activation of the dual Rac1/RhoA Rho guanine nucleotide exchange factor trio signaling pathway, which is directly linked to barrier permeability.^73^

TGF-*β* is secreted by both ECs, pericytes, astrocytes, and microglia and binds primarily to TGFBR2 (and to a lesser extent activin receptor-like kinase 5). It is integral to pericyte recruitment and differentiation, as well as upregulation of TJ genes via suppressor of mothers against decapentaplegic (SMAD) signaling. TGF-*β* derived from pericytes induces endothelial growth factors that confer resistance to hypoxia and oxidative stress, whereas endothelial-derived TGF-*β* maintains pericyte quiescence and limits proliferation.^67^^,^^74^^,^^75^

MFSD2A is expressed in brain ECs and regulates vesicular transcytosis. MFSD2A expression in ECs is modulated by pericyte coverage, such that when pericyte number decreases, MFSD2A expression also decreases, resulting in increased vesicular trafficking and subsequent BBB leakage (Fig. 1).^76^

Ayloo et al^77^ demonstrated that pericyte-secreted vitronectin, an extracellular matrix protein, binds to integrin *α*v*β*5 on ECs to actively suppress vesicular trafficking and maintain barrier integrity. Genetic ablation of vitronectin in a mouse model resulted in increased transcytosis and BBB leakage in both the retina and cerebellum, without affecting TJ proteins.^77^

Interactions between microglia and vascular cells are also crucial for BBB integrity. Direct contact between vessel-associated microglias and ECs has been observed in vivo during systemic inflammation, triggered by endothelial secretion of chemokine CCL5.^48^ Microglia have also been shown in vitro to regulate endothelial TJ protein expression, including ZO-1.^78^ Moreover, subsets of microglia associate directly with pericytes, likely also contributing to BBB stability given the importance of pericytes to this process.^79^ Interestingly, recent work using microglial depletion via colony-stimulating factor 1 receptor inhibitor PLX5622 suggested that BBB integrity may not depend entirely on microglia-vascular interactions.^80^ Although BBB integrity was maintained in the absence of microglia, the study noted potential off-target effects of PLX5622, including impacts on endothelial metabolism, which may influence these conclusions.

Oligodendrocyte precursor cells (OPCs) also interact with ECs via the basal lamina to preserve BBB integrity. The importance of OPC-EC interactions has been demonstrated both in vitro and in neonatal mice.^81^^,^^82^ Seo et al^82^ showed that OPC-derived TGF-b1 increases TJ protein expression in ECs, thereby supporting BBB function. Mature oligodendrocyte-endothelial associations have also been identified in vivo, with oligodendrocytes contacting the BBB basal lamina.^55^ Consistent with this, oligodendrocyte-derived laminin has been shown to be indispensable for BBB integrity by regulating transcellular transport in ECs.^57^

### B Regulation of cerebral blood flow

**Fig. 2:**
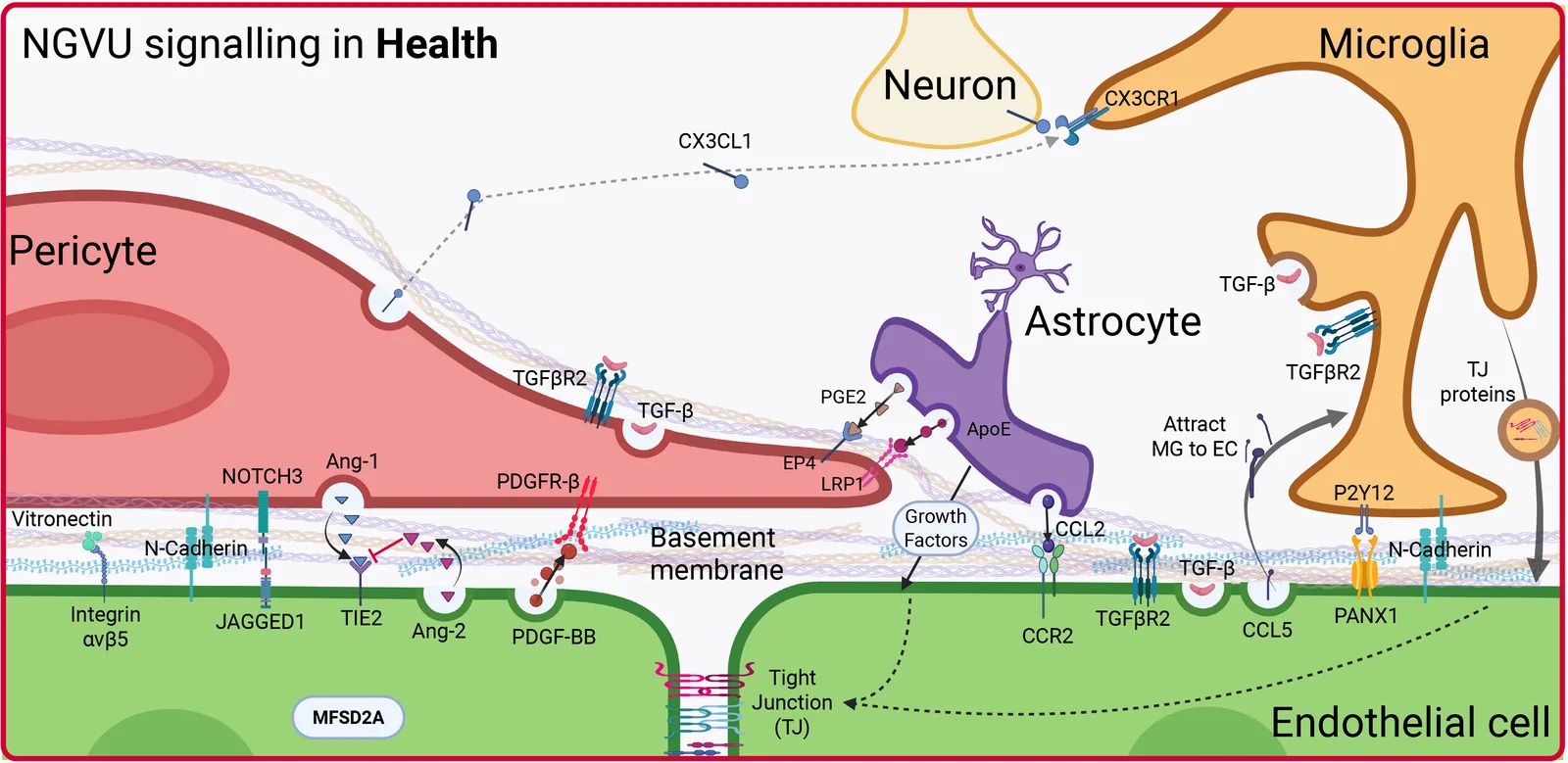
NGVU signaling in health. Schematic illustration of homeostatic signaling interactions among ECs, pericytes, astrocytes, neurons, and microglia (MG) within the NGVU, highlighting pathways that support BBB integrity and vascular stability. Endothelial–pericyte communication is shown through PDGF-BB/PDGFR-*β*, angiopoietin-1 (Ang-1)/TIE2, NOTCH3/JAGGED1, N-cadherin, and vitronectin-integrin *α*v*β*5 signaling, with MFSD2A and TJs indicated as key endothelial features associated with barrier maintenance. Astrocytes are depicted as providing trophic and regulatory inputs to the vasculature through growth factors, apolipoprotein E (ApoE)/LRP1-associated signaling, prostaglandin E2 (PGE2)/prostaglandin E receptor 4 (EP4) signaling, and CCL2/C-C chemokine receptor type 2 (CCR2)-associated communication. Neuron-to-microglia signaling is represented by CX3CL1–CX3CR1 interactions, whereas microglia–vascular crosstalk includes TGF-*β*/TGF*β*R2, CCL5-mediated recruitment toward ECs, P2Y12, and pannexin-1 signaling, regulation of N-cadherin, and modulation of endothelial TJ proteins. Together, these coordinated multicellular interactions illustrate how the healthy NGVU maintains cerebrovascular homeostasis, intercellular communication, and BBB function. Created with BioRender.com.

As discussed previously, glial cells are now recognized as important regulators of CBF, although the precise mechanisms by which they exert this control remain incompletely understood. These effects likely involve interactions between glia and pericytes, key vascular modulators of capillary diameter and flow. A central function of astrocytes is the regulation of neurovascular coupling, the process by which neuronal activity triggers local changes in CBF. Astrocyte endfeet ensheathe nearly the entire cerebral microvasculature, serving as a bridge between synaptic activity and the delivery of oxygen and glucose to meet the metabolic demands of neural tissue.^83^^,^^84^ During neuronal activation, astrocytes detect elevated levels of extracellular potassium and neurotransmitters, leading to increases in intracellular calcium. This cascade triggers the release of vasoactive substances, including prostaglandin E2 via cyclooxygenase-1, and epoxyeicosatrienoic acids via cytochrome P450 epoxygenases, promoting vasodilation and increased blood flow.^85^^,^^86^ Astrocyte-pericyte communication has been demonstrated by Mishra et al,^87^ who showed that astrocyte-derived prostaglandin E2 binds prostaglandin E receptor 4 receptors on pericytes in response to neuronal activity, inducing pericyte relaxation and increasing capillary diameter. The observed physical interactions between microglia and pericytes^47^^,^^79^ also raise the possibility that microglia may influence blood flow in coordination with pericytes, a hypothesis warranting further investigation. Supporting this, Császár et al^47^ found that microglia form direct contacts with both endothelial and smooth muscle cells during neurovascular coupling, modulating CBF via purinergic signaling, via P2RY12, suggesting that diverse glial-vascular interactions underlie cerebral perfusion regulation. Together, these findings highlight the complex and multifaceted nature of glial-vascular communication in the control of CBF (Fig. 2).

Although microglia are traditionally viewed as the primary immune sentinels of the CNS, growing evidence suggests that vascular cells also play key roles in CNS immune responses, including through direct interactions with microglia.^88^

### C Neuroinflammation and immune regulation

ECs may orchestrate communication between the CNS and the peripheral immune system during neuroinflammation. For instance, systemic bacterial infection in mice activates microglia, which release proinflammatory cytokines that act on ECs to induce adhesion molecule expression, facilitating peripheral immune cell entry into the CNS.^89^^,^^90^ Supporting this, Haruwaka et al^48^ demonstrated that endothelial secretion of CCL5 promotes microglial migration toward blood vessels during systemic inflammation.

Beyond ECs, pericytes have also emerged as important regulators of neuroinflammation. Pericyte-microglia interactions during systemic inflammation suggest that pericytes may act as immune sensors themselves.^35^ Similar to ECs, pericytes can modulate microglial activity by secreting proinflammatory cytokines such as CCL2, which enhance microglial activation.91., 92., 93., 94. Conversely, they can also release anti-inflammatory mediators that promote a neuroprotective microglial phenotype.^51^^,^95., 96., 97.

In addition to these roles, pericytes in the embryonic brain support microglial survival and proliferation,^98^ suggesting that their influence on microglial function extends across both developmental and neuroinflammatory contexts (Fig. 2).

## IV Glial–vascular interactions in neurodegenerative diseases

**Fig. 3:**
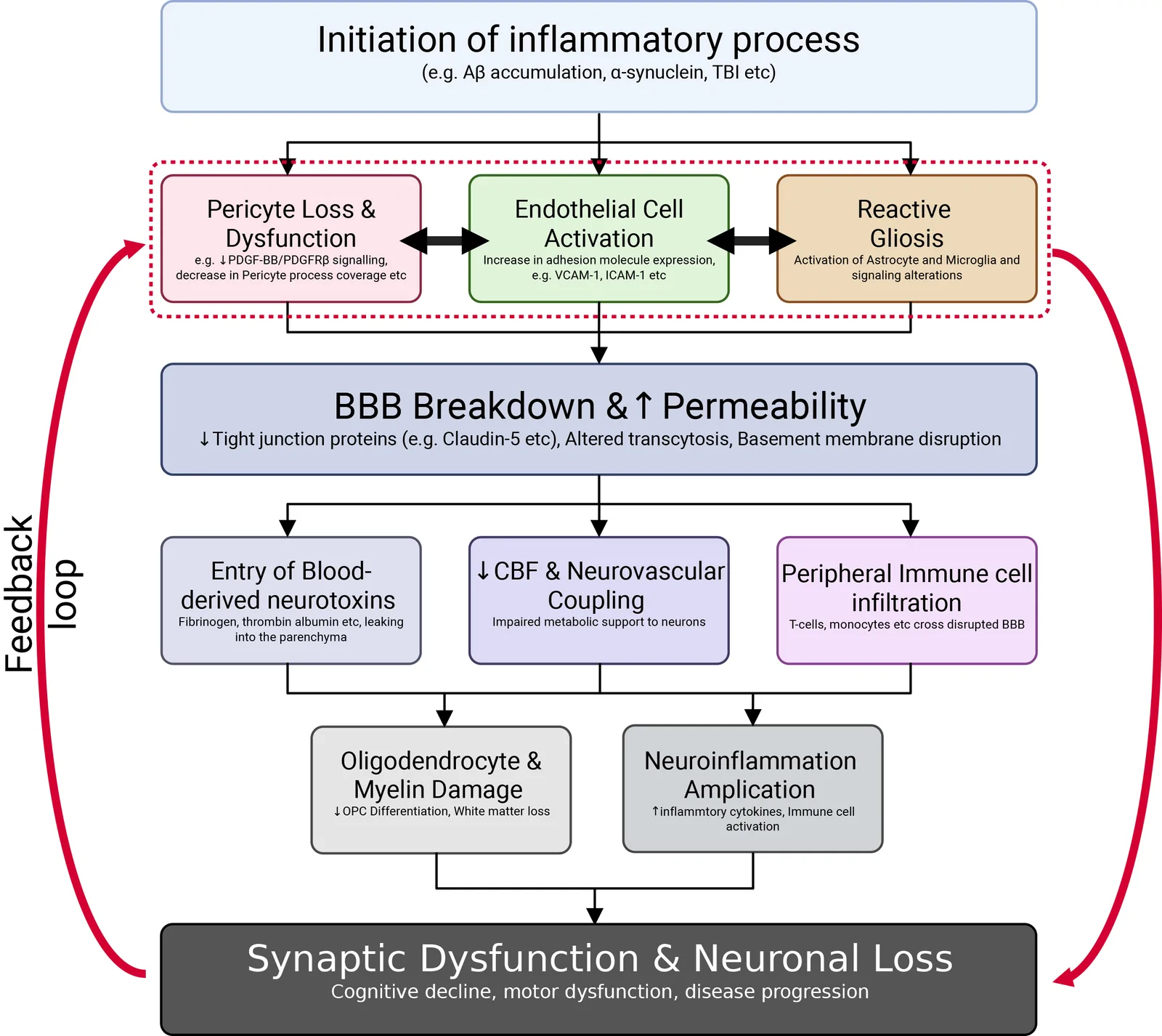
A convergent cascade of NGVU dysfunction across NDD. Schematic overview of the shared pathological mechanisms disrupting the NGVU across neurodegenerative conditions. Disease-specific triggers, including A*β* accumulation, *α*-synuclein aggregation, or ischemic insults, initiate 3 parallel upstream events: pericyte loss and dysfunction (↓ PDGF-BB/PDGFR*β* signaling), EC activation (↑ vascular cell adhesion molecule 1, ICAM-1), and reactive gliosis (astrocyte and microglial activation). These converge on BBB breakdown and increased permeability, characterized by loss of TJ proteins (claudin-5, occludin, and ZO-1), altered transcytosis, and basement membrane disruption. Downstream consequences include entry of blood-derived neurotoxins into the parenchyma, impaired CBF, and peripheral immune cell infiltration, driving neuroinflammation amplification and oligodendrocyte/myelin damage. These processes ultimately culminate in synaptic dysfunction and neuronal loss. A neuroinflammation BBB breakdown feedback loop perpetuates disease progression. Disease-specific glial–vascular mechanisms are illustrated in Fig. 4. TBI, traumatic brain injury.

Having established the key cellular interactions that maintain NGVU homeostasis, we next consider how these interactions are disrupted across a range of neurodegenerative conditions. Although the molecular triggers differ between diseases, from amyloid-*β* (A*β*) accumulation in AD and *α*-synuclein aggregation in PD, to vascular hypoperfusion in cSVD and immune-mediated demyelination in MS, a convergent cascade of NGVU dysfunction can be identified across conditions. This typically involves pericyte loss and dysfunction, EC activation, and reactive gliosis, which together drive BBB breakdown, neuroinflammation, impaired CBF, and ultimately synaptic dysfunction and neuronal loss (Fig. 3). The disease-specific glial-vascular interactions underlying each condition are discussed in the subsections below.

### A Alzheimer disease

AD has long been defined pathologically by the accumulation of A*β* and tau aggregates, as well as by loss of brain volume and cognitive dysfunction. However, growing evidence indicates that both glial and vascular dysfunctions are also central features of AD pathogenesis.^14^^,^^99^ Among the vascular abnormalities observed in AD are pericyte loss and BBB breakdown, both of which have been shown to precede and/or be associated with cognitive decline, indicating a key role for vascular dysfunction in disease progression.^28^^,^^63^^,^^100^ This causative role is further supported by studies showing that AD genetic risk variants are associated with vascular cell dysfunction and pathology.^19^^,^^63^^,^^101^

**Fig. 4:**
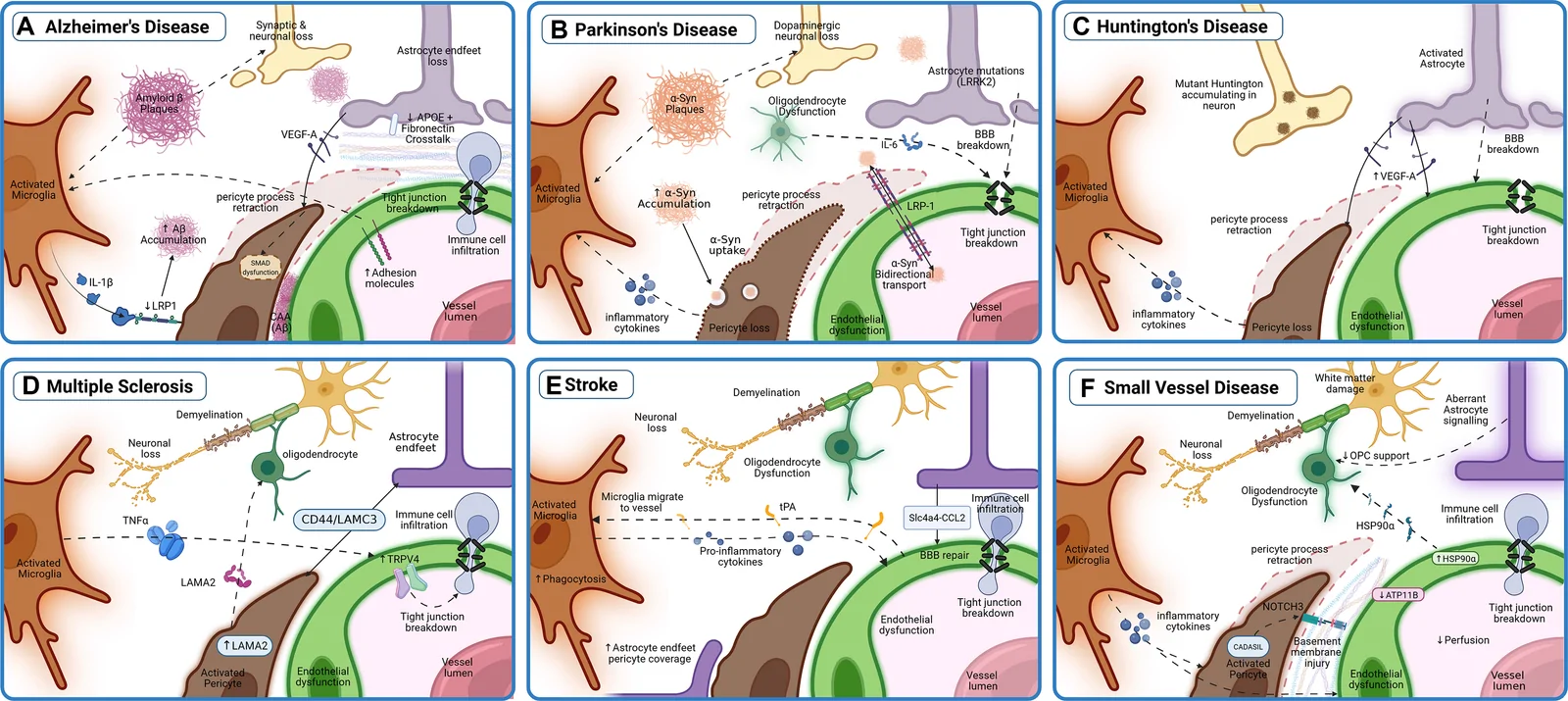
Disease-specific glial–vascular interactions across neurodegenerative conditions. Schematic overview of pathological glial–vascular mechanisms disrupting the NGVU across 6 neurological conditions. Each panel shares a common cellular architecture of activated microglia, reactive astrocytes, oligodendrocytes, pericytes, and ECs. Dashed arrows indicate disrupted or pathological signaling. Pericyte process retraction, TJ breakdown, and endothelial dysfunction are shared downstream consequences across all conditions. (a) Alzheimer disease. A*β* accumulation drives microglial interleukin-1*β* (IL-1*β*) secretion, suppressing pericyte LRP1 and impairing A*β* clearance. Dysregulated astrocytic VEGF-A-SMAD signaling and loss of fibronectin-mediated astrocyte–EC crosstalk promote BBB breakdown, immune cell infiltration, and synaptic and neuronal loss. Perivascular A*β* deposition (cerebral amyloid angiopathy [CAA]) further compromises vascular integrity. (b) Parkinson disease. *α*-Synuclein aggregation drives pericyte-derived inflammatory cytokine release and microglial activation. LRRK2-mutant astrocytes impair BBB function and vessel formation, whereas oligodendrocyte-derived interleukin-6 (IL-6) promotes endothelial dysfunction. *α*-Synuclein undergoes bidirectional LRP1-mediated BBB transport, potentially facilitating disease propagation. (c) Huntington disease. Mutant huntingtin accumulation drives microglial activation and pericyte loss. Reactive astrocyte upregulation of VEGF-A disrupts astrocyte–pericyte and astrocyte–EC signaling, promoting BBB breakdown and endothelial dysfunction. (d) Multiple sclerosis. Immune cell infiltration across a disrupted BBB is driven by ↑ transient receptor potential vanilloid-type 4 (TRPV4) expression and TJ breakdown. Microglial tumor necrosis factor-*α* (TNF*α*) propagates neuroinflammation. Pericyte-derived laminin subunit *α*2 (LAMA2) supports oligodendrocyte remyelination, whereas disrupted astrocyte–vascular crosstalk via CD44/laminin subunit gamma-3 (LAMC3) contributes to sustained BBB dysfunction. (e) Stroke. Microglial migration to the vessel wall and proinflammatory cytokine secretion perpetuate BBB disruption. Astrocytes upregulate endfoot-pericyte coverage via the Slc4a4–CCL2 axis in an attempted repair response. Tissue plasminogen activator (tPA) contributes to endothelial activation, alongside prominent oligodendrocyte dysfunction and demyelination. (f) Cerebral small vessel disease. NOTCH3 mutations (CADASIL) impair pericyte and VSMC function, driving basement membrane injury and reduced perfusion. Dysregulated heat shock protein 90*α* (HSP90*α*) and ↓ ATP11B contribute to endothelial dysfunction. Oligodendrocyte dysfunction and reduced OPC support promote demyelination and white matter damage. Created with BioRender.com.

Genomic studies have also strongly implicated microglia as another key contributor and driver of AD pathology.^102^ Indeed, alterations in the transcriptomic and phenotypic profiles of both microglia and vascular cell types have been widely reported in patients with AD.^13^^,^^103^ Given their individual contributions to disease progression, the interactions between microglia and vascular cells have emerged as an important and increasingly studied aspect of AD pathophysiology, warranting further investigation (Fig. 4a).

Recent studies using preclinical AD models have begun to uncover how microglia-EC interactions are altered in AD. For instance, Shabestari et al^104^ observed widespread transcriptomic alterations in ECs and exacerbated vascular pathology in 5xFAD mice lacking microglia. Similarly, Horibe et al^105^ found that endothelial senescence, genetically induced in APP/PS1 mice, a trait also reported in patients with AD, was associated with impaired microglial functions such as phagocytosis of A*β* plaques. Other animal studies investigating AD risk factors, including ageing, have also observed altered microglia-EC interactions. For example, Vandal et al^106^ identified increased expression of microglial activation markers in mice with reduced CD2-associated protein, a protein enriched in brain ECs and associated with AD risk. Yousef et al^89^ found that hippocampal ECs in aged mice expressed high levels of vascular cell adhesion molecule 1, a change linked to increased microglial reactivity (Fig. 4a). In human AD postmortem brain tissue, various snRNAseq studies have consistently found alterations in immune-related genes in ECs,^19^^,^^101^^,^^103^ likely influenced by microglial-EC crosstalk given microglia’s central role in innate immunity. Similar gene expression profiles have been observed in aged mice.^107^

Microglia–pericyte interactions are also increasingly implicated in AD. Using snRNAseq data to predict ligand–receptor signaling, Sun et al^108^ reported a notable upregulation of microglia–pericyte and microglia-capillary EC interactions in AD postmortem tissue. Morris et al^79^ identified a subset of pericyte-associated microglia that directly contact pericytes in the CNS, and observed a reduction of these pericyte-associated microglia in the superior frontal gyrus of patients with AD, suggesting a potential contribution to neurovascular dysfunction. Such interactions may influence the pathways involved in response to amyloid pathology, with Nortley et al^109^ showing that A*β* induces reactive oxygen species production in both pericytes and microglia in human brain slices. Similarly, Quan et al^110^ found that microglial secretion of the proinflammatory cytokine interleukin-1*β* reduces pericyte expression of LRP1, a receptor involved in A*β* clearance.^111^

Astrocytes also display transcriptomic alterations in AD.^112^ As for microglia, emerging evidence suggests that astrocyte interactions with vascular cells may also be disrupted. snRNAseq analyses implicate dysregulated astrocyte-EC and astrocyte-pericyte communication in AD.^101^^,^^108^^,^^113^^,^^114^ For example, İş et al^113^ identified altered VEGF-A-SMAD3 signaling between astrocytes and pericytes in AD postmortem tissue, with these findings validated in human induced pluripotent stem cell (iPSC)-derived models and zebrafish, where they were shown to influence BBB integrity. A*β* pathology also appears to alter astrocyte–vascular signaling; Baringer et al^115^ demonstrated that iPSC-derived astrocytes exposed to A*β* modified iron transport in cocultured ECs. Moreover, genetic risk factors such as the apolipoprotein E *ε*4 (APOE4) allele also modulate astrocyte-vascular dynamics. Recent studies show that APOE4 and A*β* drive fibronectin accumulation, which disrupts astrocyte-EC crosstalk.^116^ Prior work also implicated astrocyte-derived APOE4 as a driver of BBB impairment.^117^Together, these findings underscore the importance of continued investigation into astrocyte-vascular interactions in the context of AD risk and pathology (Fig. 4a).

### B Parkinson disease

PD is another common neurodegenerative disorder, primarily caused by the progressive loss of dopaminergic neurons in the substantia nigra.^90^ Numerous clinical studies have shown that this neuronal loss is often accompanied by vascular pathology, including BBB alterations and pericyte loss in human subjects, which may together contribute to cognitive decline.^103^^,^^118^ Other pathological hallmarks of PD, such as *α-*synuclein aggregates, also appear to be associated with vascular dysfunction, with recent work showing that these aggregates can be taken up by both vascular and glial cell types.118., 119., 120. Glial dysfunction has also been implicated in PD, and as in AD, appears to be linked to genetic risk factors.^121^ As such, glial-vascular interactions represent an important yet underexplored avenue for further investigation in PD pathogenesis, although considerably less is known about how these interactions are disrupted in PD compared with AD.

Some insights into glial-vascular crosstalk in PD have been gained from both in vitro and in vivo studies of vascular dysfunction. Dohgu et al^122^ found that *α*-synuclein exposure causes rat brain pericytes to release proinflammatory cytokines known to promote microglial activation.^122^ In a brain-chip model, de Rus Jacquet et al^123^ reported that proinflammatory astrocytes harboring a PD-associated LRRK2 G2019S mutation exhibited impaired BBB function and vessel formation, effects that could be rescued by blocking the mitogen-activated protein kinase kinases 1 and 2 (MEK1/2) signaling pathway, highlighting a role for inflammation-mediated astrocyte-vascular dysfunction in PD (Fig. 4b). In vivo, Elabi et al^124^ found that inducing diabetes, a common PD risk factor, in a PD animal model reduced pericyte–microglial interactions and exacerbated vascular pathology.

Additional evidence comes from omics-based studies. snRNAseq analysis of PD postmortem tissue by Wang et al^11^ identified ECs and pericytes as among the most transcriptionally altered cell types, with numerous differentially expressed genes between cases and controls. Subsequent cell-cell communication analysis indicated dysregulated signaling between microglia, pericytes, and ECs. However, to date, no study has performed vascular-enriched snRNAseq on PD brain tissue, an approach that has provided deeper insight into glial-vascular crosstalk in AD research.^101^

PD genetic risk has also been linked to oligodendrocytes, which in mouse models can drive neuroinflammation and neurodegeneration via cytokine secretion, including interleukin-6.^125^^,^^126^ However, whether and how oligodendrocyte-vascular interactions are altered in PD remains unknown. This question is particularly pertinent, as interleukin-6 is known to directly affect ECs,^127^ potentially linking oligodendrocyte activity to vascular dysfunction in PD.

Collectively, the available evidence points to a disrupted NGVU in PD, with pericyte-derived inflammation, dysfunction of astrocyte-vascular communication with ECs and pericytes, and transcriptomic alterations across multiple cell types, all of which converge to compromise the BBB. Mechanistically, although early alterations in the NGVU in PD may be subtle, *α*-synuclein appears to be a key upstream trigger, activating pericytes to release proinflammatory cytokines that increase endothelial permeability,^122^ whereas *α*-synuclein have also been shown to directly impair BBB function in a human brain-chip model incorporating dopaminergic neurons, and the cells of the NGVU.^128^ There is also evidence that *α*-synuclein can cross the BBB bidirectionally via LRP1-mediated transport, with inflammation appearing to enhance its entry into the brain (Fig. 4b),^129^ a mechanism potentially relevant to disease propagation. Downstream, hemodynamic consequences of NGVU disruption are increasingly apparent: neuroimaging studies have reported reduced cortical perfusion and altered cerebrovascular blood flow that may correlate with both motor and cognitive symptom severity in patients with PD.^130^^,^^131^ Whether these vascular changes are causal or secondary to neurodegeneration remains unresolved.^132^ The precise glial-vascular signaling events driving these hemodynamic alterations at the cellular level remain poorly defined, and no study has yet applied vascular-enriched transcriptomic approaches to PD brain tissue an approach that, as discussed above, has provided valuable mechanistic insight in AD.^101^

Many questions are still to be answered, including how *α*-synuclein pathology feeds back onto glial-vascular communication at the cellular level, whether vascular dysfunction precedes neuroinflammation in PD, and whether targeted transcriptomic strategies could identify the specific disrupted pathways in this disease (Fig. 4b).

### C Huntington disease

HD is an autosomal dominant neurodegenerative disorder caused by a cytosine-adenine-guanine trinucleotide repeat expansion in the huntingtin (*HTT*) gene.^133^ Both glial and vascular dysfunctions are thought to contribute significantly to HD pathogenesis, with vascular abnormalities appearing early in disease progression.^134^^,^^135^ Evidence for vascular dysfunction has emerged from snRNAseq studies. Notably, Garcia et al^136^ performed extensive profiling of vascular cell types in HD postmortem brain tissue, identifying altered gene expression across ECs, pericytes, and VSMCs. In addition to vascular cell dysfunction, the authors observed upregulation of immune response-related genes in ECs, astrocytes, and microglia, such as findings reported in AD studies. Protein analysis in mouse tissue confirmed upregulation of protein kinase R, one of the implicated genes, specifically in glial cells in contact with blood vessels. Moreover, Garcia et al^136^ noted enrichment of vascular-associated genes within subclusters of astrocytes and microglia in their snRNAseq dataset. Taken together, these findings strongly implicate disrupted glial-vascular crosstalk in late-stage HD.

Further evidence for altered glial-vascular crosstalk has been established in vitro. Hsiao et al^137^ found that astrocytes from HD mice exhibit a proinflammatory phenotype and can modulate both EC proliferation and pericyte survival. These effects were attributed to increased astrocytic expression of VEGF-A in both HD mice and human patients, which may impact BBB integrity. This finding is of particular interest in light of similar alterations in astrocytic VEGF-A signaling observed in AD models and human patients,^113^ suggesting VEGF-A dysregulation may be a common mechanism of glial-vascular dysfunction across NDDs. The work by Hsiao et al^137^ may also relate to the loss of pericytes observed in both HD mouse model and human tissue,^138^ which appears to precede cognitive decline. In support of this, Linville et al^139^ reported that iPSC-derived ECs from patients with HD exhibit altered VEGF responsiveness, indicating that endothelial dysfunction may arise as a downstream consequence of dysregulated astrocytic VEGF-A signaling.

Additional iPSC-based studies have highlighted further mechanisms of glial–vascular disruption. Hernandez et al^140^ reported perturbations at the extracellular matrix-integrin interface between astrocytes and ECs, suggesting a possible structural basis for impaired cell-cell communication in HD. Finally, as observed in PD, aberrant oligodendrocyte signatures have also been identified in both patients with HD and preclinical models (Fig. 4c).^141^^,^^142^ However, whether these alterations impact oligodendrocyte-vascular interactions remains to be determined.

Currently, the available evidence implicates astrocytic overexpression of VEGF-A as a central mechanistic axis linking glial dysfunction to vascular pathology in HD, with downstream consequences including aberrant angiogenesis, pericyte loss, and impaired vascular reactivity demonstrated in both mouse models and patient tissue.^133^ Chronic VEGF-A elevation appears paradoxically detrimental: rather than driving compensatory angiogenesis, it promotes the formation of morphologically abnormal vessels with reduced diameter and compromised perfusion capacity despite increased vessel density.^143^ This is of particular interest given that similar dysregulation of astrocytic VEGF-A signaling has been observed in AD,^113^ suggesting this may represent a shared mechanism of glial-vascular dysfunction across NDDs. Pericyte loss, which may be exacerbated by aberrant VEGF-A signaling, appears to precede cognitive decline in HD^133^^,^^134^ and is further supported by iPSC-based evidence demonstrating altered VEGF responsiveness in patient with HD-derived ECs,^135^ indicating that endothelial dysfunction may arise as a downstream consequence of dysregulated glial–vascular signaling (Fig. 4c). However, critical mechanistic questions remain unresolved: it is unclear how early these NGVU changes arise relative to neuronal loss, whether pericyte depletion feeds back to further dysregulate glial activity, and whether oligodendrocyte-vascular interactions, as yet unstudied in HD, contribute to the white matter changes observed in patients.

### D Multiple sclerosis

Multiple sclerosis is both a white matter and neurodegenerative disorder in which vascular dysfunction also appears to be an inherent characteristic.^144^ Vascular pathologies reported in patients with MS include EC dysfunction and reduced blood flow, with an disrupted BBB perpetuating immune cell infiltration into the CNS.^145^ Aberrant microglial, astrocytic, and oligodendrocytic profiles have also been reported in patients, and are thought to be key drivers of pathology such as demyelination.^146^^,^^147^

Given the prominent white matter pathology in MS, there is particular interest in profiling the primary myelin-producing cells, oligodendrocytes, and their interactions with other cell types in the CNS. Indeed, oligodendrocyte-vascular cell interactions are increasingly being implicated in the pathophysiology of MS. Notably, pericytes have been shown to respond to demyelination and promote OPC differentiation and subsequent remyelination by secreting laminin subunit *α*2 (Fig. 4d).^148^ Interestingly, laminin subunit *α*2 expression has also been shown to increase in lesion tissue from patients with relapsing-remitting MS.^149^ Aberrant oligodendrocyte-endothelial interactions in MS were also reported by Niu et al,^150^ who found in vivo that perivascular migration of OPCs in lesions disrupted the BBB and EC TJ integrity, subsequently exacerbating neuroinflammation. These findings highlight the possibility that oligodendrocyte-vascular interactions are simultaneously associated with white matter and vascular pathology in MS.

Astrocyte- and microglia-vascular crosstalk may also be an important contributor to MS pathogenesis. Interactions of interest include those between microglia and ECs, as suggested by evidence from postmortem tissue. This includes an increased number of ECs identified in MS lesion microglial modules, which were found in the same study to be associated with more severe MS pathology.^151^ Higher numbers of vascular cells were also identified through spatial transcriptomics in the brain immune-infiltrated regions of the MS mouse model experimental autoimmune encephalomyelitis.^152^ This region was also populated by disease-associated glia (astrocytes, microglia, and oligodendrocytes), which the authors of the study suggested may require the brain vasculature to facilitate cytokine secretion. Microglia–EC interactions have also been directly linked to BBB dysfunction in MS, with microglial-derived tumor necrosis factor-*α* shown in vitro to modulate expression of the transient receptor potential vanilloid-type 4 ion channel, which shows increased expression in MS lesions from postmortem tissue (Fig. 4d).^153^ Importantly, transient receptor potential vanilloid-type 4 was found in the same work to downregulate expression of EC TJ proteins and exacerbate BBB rupture in an inflammatory context. Roles for astrocyte–vascular crosstalk were further postulated in another recent spatial transcriptomics study using human MS lesion tissue.^154^ This study identified several astrocyte-vascular interactions that appear to be enriched in MS lesions, such as those between astrocytic CD44 and pericyte laminin subunit gamma-3 proteins, the latter of which is associated with processes such as angiogenesis.^155^

### E Stroke and small vessel disease

Although not classified as neurodegenerative disorders, stroke, and cSVD are significant risk factors for neurodegeneration, notably AD.156., 157., 158. Importantly, altered glial-vascular crosstalk has been implicated in both of these disorders. Given their strong associations with risk of diseases such as AD, these interactions may overlap with the earliest phases of disease progression.

In the context of stroke, numerous studies have observed altered microglia–EC interactions that may be both beneficial and detrimental to pathology. A recent study found in a thrombotic stroke mouse model that EC-derived tissue-type plasminogen activator facilitates recruitment of microglia to blood vessels soon after stroke induction and helps preserve BBB integrity.^159^ Conversely, in other stroke models, microglia have been shown to potentiate vascular pathology and promote EC death through processes such as the release of proinflammatory cytokines and phagocytosis.160., 161., 162. Microglia-pericyte interactions, on the other hand, may be more protective in nature, with a combined transplant of microglia and pericytes in a mouse stroke model found to attenuate neuroinflammation and improve cognitive function.^163^ Pericytes have also been found to acquire a microglial-like phenotype during stroke,^39^^,^^164^^,^^165^ which may involve secretion of paracrine factors from other cell types to facilitate this transition.

Furthermore, several studies have indicated protective roles for astrocyte-EC axis in stroke. Indeed, Ye et al^166^ reported that the astrocyte-endothelial Slc4a4-CCL2 axis mediates BBB repair after stroke (Fig. 4e). Similarly, Williamson et al^167^ also found that reactive astrocytes promote BBB repair poststroke, with astrocyte ablation leading to reduced pericyte coverage of vessels. Vascular cells may additionally support astrocyte function and survival during stroke, with in vitro work showing mitochondrial transfer from pericytes and ECs to astrocytes, which prevented their apoptosis in ischemia/reperfusion conditions.

Comparatively, far less is known about glial-vascular interactions in cSVD, although one such interaction that is of increasing interest is the oligodendrocyte-endothelial axis, which may relate to the white matter pathology seen in clinical studies.^168^ Rajani and Williams^59^ found in a rat model of cSVD that dysfunctional ECs secreted elevated levels of heat shock protein 90*α*, which impaired oligodendrocyte differentiation and myelination.^59^ Follow-up work from the same group also demonstrated that loss of ATP11B expression in ECs impeded oligodendrocyte differentiation and led to white matter abnormalities and cSVD-like pathology in rats.^169^ Increased microglial activation has also been observed in cSVD white matter pathology,^170^ although the impact of this on vascular cell profiles, if any, remains unknown. Furthermore, aberrant astrocyte signaling has been noted in a diet-based model of cSVD,^171^ but whether this influences astrocyte-vascular interactions has not yet been deciphered. The continued development of effective animal models of cSVD, which have previously been lacking, will hopefully further pinpoint how glial-vascular crosstalk is perturbed in this disease. This includes alterations in interactions with pericytes, which clinical studies also suggest are disrupted in cSVD (Fig. 4f).^172^

Endothelial dysfunction and BBB leakage are well established as central features of cSVD pathogenesis,^173^ yet the full mechanistic understanding of how the cells of the NGVU interact to drive or perpetuate these changes remains considerably less characterized than in other diseases discussed in this review. Notably, recent epigenomic profiling of brain neurovascular cell types has revealed that genetic risk of small vessel disease is enriched across multiple NGVU components, including astrocytes and ECs, further underscoring the need for integrated, multicell-type approaches.^174^ Emerging evidence suggests a bidirectional feedback loop in which initial glial activation, likely triggered by hypoperfusion and/or ischemia, releases inflammatory mediators that injure the endothelial and pericyte basement membrane complex, further increasing BBB permeability and amplifying neuroinflammation.^175^ Disruption of the NGVU appears particularly consequential for white matter integrity: BBB leakage activates astrocytes and triggers microglial reactivity in a manner that suppresses OPC differentiation and maturation, thereby compounding myelin loss.^176^ This is consistent with the established oligodendrocyte-endothelial axis in cSVD,^59^ and further supported by the observation that aberrant EC dysfunction directly impedes oligodendrocyte differentiation and produces white matter abnormalities in preclinical cSVD models.^39^ Critical mechanistic questions remain unresolved: whether glial–vascular crosstalk differs across cSVD subtype, such as cerebral autosomal dominant arteriopathy with subcortical infarcts and leukoencephalopathy versus sporadic cSVD, what initiates the transition from early pericyte dysfunction to overt glial reactivity, and whether targeting glial-endothelial signaling axes could modify the progressive white matter damage that underlies cognitive decline (Fig. 4, e and f).

## V Future directions

Based on the findings from the body of literature discussed in this review, glial-vascular interactions are most likely both a “friend” and a “foe” to the CNS. They are crucial for supporting the various functions of the NGVU across the lifespan but also show widespread alterations across multiple neurodegenerative disorders and associated risk factors. Together, these observations highlight glial-vascular interactions as a promising therapeutic target for diverse CNS disorders. However, such therapeutic advancements require a deeper understanding of the exact nature and impact of this altered crosstalk on disease pathogenesis. We now outline key knowledge gaps concerning these interactions and propose future research directions necessary to drive therapeutic development.

### A Glia and vascular cell heterogeneity

One important aspect that has often been overlooked when assessing glial-vascular interactions is the heterogeneity of glial and vascular cells in both health and disease. Altered glial heterogeneity is a characteristic feature of many of the diseases discussed in this review. For instance, the presence of a subset of microglia known as “disease-associated microglia” has been documented in models of neurodegenerative disorders such as AD and MS.^177^ Distinct alterations in the gene signatures of subtypes of vascular cells, such as arterial-venous EC subsets, are also beginning to be noted in diseases such as AD.^13^^,^^19^^,^^108^^,^^178^

This raises an important question for the field: is glial-vascular communication particularly disrupted between specific cellular subpopulations in these disorders? Identifying subtypes in these diseases, however, has proved challenging in the case of vascular cells, which typically represent a small fraction of cells in omics datasets such as those generated from snRNAseq, because of the depletion of vascular nuclei for still-unclear reasons.^19^ This challenge can be addressed by applying vascular-enrichment protocols, which enable extraction and sequencing of sufficient vascular cells to identify subtypes such as ECs along the vascular bed.^19^ Such approaches should continue to be implemented in future omics studies investigating the role of glial–vascular crosstalk in neurodegenerative disorders. These studies should aim to capture both glial and vascular cells at sufficient depth and power before downstream analyses of intercellular communication.

### B Tools to assess glial–vascular crosstalk

In recent years, several powerful bioinformatic tools have been developed, such as NicheNet^179^ and CellChat,^180^ that can infer alterations in intercellular communication using ligand–receptor interaction databases in multicellular single-cell and single-nucleus datasets. However, these tools are entirely predictive in nature, and their accuracy is difficult to evaluate because of the absence of a ground truth in the field.^181^

Future efforts to dissect glial-vascular crosstalk may benefit from complementary experimental approaches. This may include novel sequencing technologies such as spatial proximity enabled analysis of crosstalk sequencing^182^ and rabies virus-based barcoded interaction sequencing,^183^ both of which have directly identified altered microglial-astrocyte crosstalk in preclinical models of MS. Another emerging and rapidly evolving sequencing technology that will likely advance our understanding of these interactions is spatial transcriptomics, which can infer changes in intercellular communication based on the spatial proximity of cells. Indeed, the power of this approach to decipher cell-cell communication in diseases such as AD and MS has been recently demonstrated by studies performing spatial transcriptomics on tissue from both animal models and patients.^152^^,^^184^ Next-generation spatial transcriptomic platforms offering single-cell resolution will be particularly valuable for identifying disease-relevant glial and vascular subtypes. Additionally, proteomic approaches may be especially valuable for mapping interactions involving cell types where there is a weaker correlation between gene expression and protein abundance, such as astrocytes.^185^ Indeed, proteomic tools have now been developed which can even resolve crosstalk between vascular cells and subcellular structures such as astrocyte endfeet.^186^

Although omics and computational approaches have greatly advanced our understanding of the molecular identities and predicted interactions of NGVU cell types, these techniques still need to be anchored by functional readouts that directly measure BBB integrity and hemodynamics, ideally in both human in vitro and mouse in vivo settings.

In vitro modeling of the NGVU, particularly using human cells, offers important translational value. Transendothelial electrical resistance and paracellular tracer permeability assays, which quantify the passage of fluorescent molecules such as dextran or sodium fluorescein across an endothelial monolayer, remain gold standard approaches for assessing barrier function.^187^ The power of these assays is substantially enhanced when ECs are cocultured with other NGVU cell types, including pericytes, astrocytes, and microglia, enabling assessment of glial-vascular communication under pathophysiologically relevant conditions. The use of iPSC-derived human cells further strengthens this approach by enabling isogenic comparisons, as has been demonstrated in models of both AD and PD.^188^ These in vitro models have been deployed effectively in organ-on-chip platforms that coculture multiple NGVU cell types under physiological shear stress, enabling real-time monitoring of barrier responses to inflammatory stimuli relevant to neurodegeneration.^128^

In vivo, equally powerful imaging-based approaches allow quantification of NGVU function at the whole-brain and cellular level. Dynamic contrast-enhanced magnetic resonance imaging enables noninvasive quantification of subtle BBB permeability changes across brain regions, whereas arterial spin labeling magnetic resonance imaging provides complementary measures of CBF and hemodynamic impairment.^189^ Intravital two-photon microscopy with fluorescent tracer injection further allows direct visualization of vascular leakage, pericyte dynamics, and immune cell–vessel interactions at single-cell resolution in live animals.^109^

Beyond these experimental tools, gene therapy-based strategies are increasingly being considered as a means of targeting brain ECs directly. Engineered adeno-associated virus (AAV) vectors with tropism for brain ECs, such as AAV-X1, AAV-BR1, and the recently described rAAV-miniBEND system, have demonstrated cell-type-specific gene delivery to the cerebrovasculature in animal models,^190^^,^^191^ and their application to modulate glial–vascular signaling axes in neurodegeneration represents an exciting emerging avenue. Although such approaches are not the primary focus of this review, they are briefly noted here given their therapeutic relevance to the NGVU. For a comprehensive overview of gene therapy strategies targeting brain ECs, readers are referred to recent dedicated reviews on this topic.^192^

Crucially, a major opportunity for the field lies in combining these functional assays with spatial transcriptomics or single-cell omics on the same tissue or model system, for example, pairing dynamic contrast-enhanced magnetic resonance imaging with postmortem snRNAseq in the same patient cohort, or coupling transendothelial electrical resistance measurements with spatial proximity enabled analysis of crosstalk sequencing or ligand-receptor analysis in organ-on-chip systems, thereby bridging the gap between transcriptional inference and functional consequence. Such integrated approaches will be essential for translating molecular discoveries about glial-vascular crosstalk into therapeutically meaningful targets.

### C How are glial-vascular interaction altered?

Another important question for the field, and one that remains largely unresolved, is how glial-vascular communication changes over the course of disease progression. As discussed previously, certain alterations in glial-vascular interactions have been linked to various risk factors for NDDs, including AD, suggesting that such changes may arise early in disease development.

However, much of the current evidence for altered crosstalk in NDDs comes from omics studies of postmortem tissue, which offer limited insights into the cellular and molecular dynamics of early disease stages. Preclinical disease models will therefore play an essential role in addressing this temporal dimension. This includes not only animal models but also patient-derived stem cell technologies with greater translational relevance.

One such promising system is the recently developed three-dimensional (3D) miBrain model, A patient-specific, iPSC derived cellular model, featuring the 6 major brain cell types (brain microvascular endothelial cells, pericytes, OPCs, neurons, astrocytes, and microglia-like cells) in a hydrogel. This model is of special interest because it incorporates both glial and vascular cell types, features previously lacking in other 3D systems such as organoids.^188^ Using this approach, Stanton et al^188^ demonstrated altered astrocyte-microglial crosstalk associated with the *APOE4* genetic risk variant in AD, highlighting the potential of integrated 3D human models to uncover mechanisms of glial-vascular dysfunction.

### D Development of glial–vascular targeted therapeutics

The therapeutic landscape targeting the NGVU has expanded significantly in recent years, encompassing a diverse array of interventions across various stages of development. Our review identifies 28 distinct direct therapeutic approaches that specifically target components of the NGVU for the treatment of neurodegeneration and related disorders, as detailed in Table 1.193., 194., 195., 196., 197., 198., 199., 200., 201., 202., 203., 204., 205., 206., 207., 208., 209., 210., 211., 212., 213., 214., 215., 216., 217., 218., 219., 220. Additionally, 14 specialized drug delivery systems have been developed to overcome BBB limitations (Table 2).^18^^,^221., 222., 223., 224., 225., 226., 227., 228., 229., 230., 231., 232., 233. It should be noted that both tables are illustrative rather than exhaustive (entries were selected based on the following criteria: agents with primary targets in vascular or glial components of the NGVU and at least preclinical evidence of efficacy in neurodegeneration or a related CNS indication, given the pace at which this field is advancing, readers are encouraged to consult primary literature for the most up-to-date pipeline). Among the direct therapeutics listed in Table 1, the pipeline includes 7 US Food and Drug Administration (FDA)-approved therapies, 5 agents in clinical trials, and 16 therapeutics in preclinical development. This distribution reflects both the complexity of NGVU targeting and the growing recognition of its therapeutic potential across multiple disease indications.

**Table 1:** Therapeutics targeting the NGVU

Therapeutic	Target Cell/Area	Disease Indication	Development Stage	Therapeutic Class	References
Lecanemab	Microglia, vascular amyloid	AD	FDA approved	Monoclonal antibody	Dyck et al^193^
Aducanumab	Microglia, vascular amyloid	AD	FDA approved	Monoclonal antibody	Wojtunik-Kulesza et al^194^
Donanemab	Microglia, vascular amyloid	AD	FDA approved	Monoclonal antibody	Rashad et al^195^
Natalizumab (Tysabri)	Endothelial/immune	MS	FDA approved	Monoclonal antibody	Häusler et al^196^
Bevacizumab (Avastin)	Endothelial/vascular	Brain cancer, NDD/AD	FDA approved	Monoclonal antibody	Mukherji^197^
Fingolimod (Gilenya)	Astrocytes/BBB	Multiple sclerosis	FDA approved	Small molecule	Sharma et al^198^
Teprotumumab	Endothelial (IGF-1R)	Thyroid eye disease (potential NDD)	FDA approved	Monoclonal antibody	Douglas et al^199^
MSCs	BBB/multiple	AD, PD, stroke, MS	Clinical trials	Cell/gene therapy	Isaković al^200^
Laromestrocel	Vascular, inflammation	AD, ALS	Phase 2a trial	Cell/gene therapy	Rash et al^201^
PDGF-BB	Pericytes	BBB dysfunction, Parkinson’s	Clinical trials	Growth factor	Li et al^202^
AST-004	Astrocytes/neuroprotection	Stroke, TBI, AD	Phase 1b	Small molecule	Manna et al^203^
PLX3397 (pexidartinib)	Microglia	NDD, brain cancer	Phase 1–3 trial	Small molecule	Wen et al^204^
LY3022855 (IMC-CS4)	Microglia	Cancer (potential NDD)	Phase 1	Monoclonal antibody	Autio et al^205^
Carboplatin + FUS-BBBO	BBB	Brain cancer	Phase 1/2a	Physical + chemotherapy	Carpentier et al^206^
Gantenerumab	Vascular/BBB (amyloid clearance)	AD	Phase 3 failed	Monoclonal antibody	Bateman et al^207^
Solanezumab	Vascular/BBB (amyloid clearance)	AD	Phase 3 failed	Monoclonal antibody	Doggrell^208^
AL002a (TREM2 agonist)	Microglia	AD	Phase 2 failed	Monoclonal antibody	Long et al^209^
PLX5622	Microglia	AD, MS	Preclinical	Small molecule (CSF1R inhibitor)	Green et al^210^
GW2580	Microglia	PD, MS	Preclinical	Small Molecule (CSF1R inhibitor)	Borjini et al^211^
JNJ-40346527	Microglia	AD, brain cancer	Preclinical, phase 1/2 (cancer)	Small molecule (CSF1R inhibitor)	Mancuso et al,^234^ von Tresckow et al^212^
Wnt3a/CHIR99021	BBB/endothelial	BBB dysfunction (cancer and NDD)	Preclinical	Small molecule	Law and Zheng^213^
SW033291 (15-PGDH inhibitor)	BBB, microglia/PVM	AD, TBI	Preclinical	Small molecule	Koh et al^214^
EPPS	BBB/amyloid clearance	AD	Preclinical	Small molecule	Kim et al^215^
Thalidomide	Pericytes	BBB dysfunction, Alzheimer’s	Preclinical/phase 2a	Small molecule	Decourt et al^216^
7,8-Dihydroxyflavone	Oligodendrocytes/neurons	AD, depression	Preclinical	Small molecule	Devi and Ohno^217^
LM22A-4	Oligodendrocytes/neurons	AD, HD, Rett syndrome	Preclinical	Small molecule	Kazim and Iqbal^218^
AL04	BBB/endothelial	NDD	Preclinical	Fusion protein	Panaiotov^219^
TDP6 (BDNF mimetic)	Oligodendrocytes	Demyelinating diseases	Preclinical	Peptide mimetic	Schirò et al^220^

ALS, amyotrophic lateral sclerosis; BDNF, brain-derived neurotrophic factor; CSF1R, colony-stimulating factor 1 receptor; EPPS, 4-(2-hydroxyethyl)-1-piperazinepropanesulfonic acid; FUS-BBBO, focused ultrasound-induced BBB opening; GW2580, CSF1R inhibitor; IGF-1R, insulin-like growth factor 1 receptor; LY3022855, TREM2 agonist candidate; 15-PGDH, 15-hydroxyprostaglandin dehydrogenase; PLX3397, pexidartinib (CSF1R inhibitor); PLX5622, CSF1R inhibitor (microglial depletion agent); PVM, perivascular macrophages; TBI, traumatic brain injury; TREM2, triggering receptor expressed on myeloid cells 2.

**Table 2:** CNS drug delivery systems

Delivery System	Target Area	Disease Application	Development Stage	Key Features	References
Focused ultrasound + microbubbles	BBB	AD, PD, brain cancer	Phase 2 (AD)	Transient BBB opening	Mehta^221^
Solid lipid nanoparticles	BBB	Various NDD	Preclinical	Lipid-based drug carriers	Satapathy et al^222^
Polysorbate 80-coated nanoparticles	BBB	CNS disorders	Preclinical	Enhanced BBB permeability	Nguyen et al^223^
Polymeric nanoparticles	BBB	CNS disorders	Preclinical	Biodegradable carriers	Annu et al^224^
Dendrimers	BBB	CNS disorders	Preclinical	Branched macromolecules	Kaurav et al^225^
Metallic nanoparticles	BBB	CNS disorders	Preclinical	Metal-based carriers	Toader et al^226^
Gold nanoparticles	BBB	CNS disorders	Preclinical	Biocompatible metal carriers	Chiang et al^227^
Carbon nanotubes	BBB	CNS disorders	Preclinical	Carbon-based carriers	Wilson et al^228^
Curcumin nanoparticles	BBB/anti-inflammatory	AD	Preclinical/clinical	Anti-inflammatory delivery	Pei et al^229^
Selenium nanoparticles	BBB/neuroprotection	AD	Preclinical	Neuroprotective delivery	Vicente-Zurdo et al^230^
LPS-targeting liposomes	Microglia (TLR4)	ALS	Preclinical	Microglia-specific targeting	Wiley et al^231^
CD11b antibody nanoparticles	Microglia	Neuropathic pain	Preclinical	Antibody-conjugated targeting	Zhao et al^18^
Liposomes	BBB	CNS disorders	Preclinical	Phospholipid vesicles	Aparicio-Blanco and Torres-Suárez^232^
CLR 124/CLR 131	BBB	Brain cancer	Phase 1 trial	Carrier system	Longcor and Oliver^233^

ALS, amyotrophic lateral sclerosis; CD11b, cluster of differentiation 11b; CLR, cationic liposome-based radiotherapy; LPS, lipopolysaccharide; TLR4, Toll-like receptor 4.

#### 1 Clinical translation success stories

The field has achieved notable clinical successes, particularly with FDA-approved monoclonal antibodies targeting microglia and vascular amyloid deposits. As shown in Table 1, the recent approval of lecanemab,^193^ aducanemab,^194^ and donanemab^195^ represents the first generation of disease-modifying treatments for AD. However, their impact on the NGVU remains underexplored and may pose challenges when applied to patients’ early disease progression, when BBB integrity is largely preserved. These antiamyloid antibodies demonstrate that modulating glial-vascular interactions can yield clinically meaningful outcomes in neurodegenerative conditions, both beneficial and detrimental, as exemplified by the amyloid-related imaging abnormalities events associated with cerebral amyloid angiopathy in lecanemab-treated patients.^234^

Additional FDA-approved therapeutics demonstrate the breadth of NGVU-targeting approaches. For instance, natalizumab (Tysabri) exemplifies successful endothelial-immune targeting in MS through its inhibition of *α*4-integrin-mediated leukocyte-endothelial interactions.^197^

#### 2 Emerging physical interventions

Focused ultrasound with microbubbles has emerged as a promising NGVU-targeted therapy, advancing to phase 2 clinical trials for AD, PD, and brain cancer (Table 2).^221^^,^^236^ This technology enables transient, reversible BBB opening without inducing acute neuronal damage, representing the only currently available noninvasive and controllable BBB-opening method, although its long-term safety remains uncertain. The approach has shown promise when combined with various therapeutic agents, including the combination of carboplatin plus focused ultrasound-induced BBB opening, which has progressed to phase 1/2a trials for brain cancer (Table 1). Multiple studies have confirmed the safety and efficacy of this technique in small clinical cohorts across several indications, highlighting the growing therapeutic versatility of physical, nonpharmacological interventions.

#### 3 Novel cellular and molecular strategies

Among the most innovative strategies are cell-based and gene therapies targeting multiple NGVU components. Mesenchymal stem cells (MSCs) represent a particularly promising multitarget approach, currently in clinical trials for AD, PD, stroke, and MS (Table 1).^200^ These therapies modulate BBB function and multiple cellular components simultaneously, addressing the complex nature of NGVU dysfunction.

Recent research has demonstrated the remarkable potential of MSC therapy in AD. The phase 2a clinical trial of laromestrocel showed that allogeneic MSC therapy significantly improved clinical outcomes and reduced brain atrophy by 48.4% for total brain volume and 61.9% for left hippocampal volume compared with placebo.^237^ This represents the first successful demonstration of a cellular therapy capable of preserving brain structure in patients with AD.

Colony-stimulating factor 1 receptor inhibitors represent another promising therapeutic class targeting microglia, with agents including PLX5622, PLX3397 (pexidartinib), and GW2580 (Table 1).^204^^,^^210^^,^^211^ PLX5622 has shown robust efficacy in rapidly depleting nearly all microglia within 7 days when administered via standard rodent chow, with full repopulation occurring within several days after withdrawal. However, caution is warranted, as microglial depletion disrupts essential CNS and vascular maintenance functions, exacerbating vascular pathology in mouse models lacking microglia.^104^^,^^238^ To mitigate this, microglial depletion could be followed by replacement with “patient-corrected” microglia, an approach that rescued vascular pathology in a chimeric mouse model featuring axonal spheroids and pigmented glia.^239^

#### 4 Drug delivery systems for neurogliovascular unit targeting

The development of sophisticated nanoparticle-based delivery systems represents a major frontier in NGVU therapeutics, with 14 distinct approaches currently under investigation (Table 2). These range from neurotransmitter-derived lipidoids developed at Tufts University, described as “extremely versatile and relatively non-disruptive,” to more established platforms such as solid lipid nanoparticles, polymeric nanoparticles, and polysorbate 80-coated nanoparticles that enhance BBB permeability through receptor-mediated transcytosis.^240^

Advanced metallic and hybrid nanomaterials, including gold nanoparticles and carbon nanotubes, offer unique physicochemical properties, enabling high drug loading and controlled release. Likewise, curcumin and selenium nanoparticles exemplify dual-function formulations with both therapeutic and delivery capacities, showing promise for anti-inflammatory and neuroprotective applications in AD and related disorders.

Despite these advances, the field has experienced instructive setbacks underscoring the complexity of NGVU targeting. The phase 2 INVOKE-2 failure of the triggering receptor expressed on myeloid cells 2 agonist AL002a, despite confirmed target engagement, highlights the critical importance of timing and patient stratification. Similarly, the phase 3 failures of gantenerumab and solanezumab, which reduced amyloid burden without clinical benefit, have driven a paradigm shift toward combination therapies, biomarker-guided patient selection, and precision medicine approaches integrating direct therapeutics with advanced delivery systems.^207^

Exosomes have emerged as particularly promising BBB-crossing vehicles. These nano-sized extracellular vesicles (50–100 nm) naturally traverse the BBB via transcytosis, exhibit low immunogenicity, and can be engineered for targeted delivery. MSC-derived exosomes offer reduced adverse effects compared with MSCs themselves, whereas macrophage-derived exosomes leverage integrin lymphocyte-associated antigen-1 and endothelial intercellular adhesion molecule-1 interactions for vascular entry.

Clinical translation of delivery systems has reached notable milestones. Focused ultrasound plus microbubbles remains the most advanced physical delivery platform, enabling transient BBB opening with acceptable safety, whereas cationic liposome-based radiotherapy 124/cationic liposome-based radiotherapy 131 carrier systems have advanced to phase 1 clinical evaluation for brain cancer (Table 2).

Current research increasingly focuses on combination approaches that address the multifaceted nature of NGVU dysfunction, integrating direct therapeutics, advanced delivery systems, and precision medicine strategies guided by NGVU biomarker profiles. Given substantial patient heterogeneity in NDD, biomarker-driven stratification and optimal timing of intervention are likely to be crucial for therapeutic efficacy. At the same time, preserving homeostatic NGVU function remains essential to prevent treatment-induced toxicity.

The field continues to evolve rapidly through expanding collaboration among neuroscientists, clinicians, bioengineers, and pharmaceutical developers. Ultimately, success will depend on harmonizing mechanistic understanding with clinical translation, as reflected in the growing therapeutic landscape summarized in Tables 1 and 2, highlighting both remarkable progress and a promising horizon for NGVU-targeted therapies in neurodegeneration.

## VI Conclusion

This review highlights that glial-vascular interactions within the NGVU serve as both essential physiological mechanisms and critical pathological contributors across the spectrum of NDDs. In the healthy brain, coordinated crosstalk between glial and vascular cells maintains BBB integrity, regulates CBF, and orchestrates immune surveillance. However, in disorders such as AD, PD, HD, stroke, cSVD, and MS, these interactions become profoundly disrupted, contributing to disease onset and progression.

The inherent heterogeneity of glial and vascular cell populations adds further complexity, with evidence suggesting that specific cellular subtypes may be differentially affected across disease contexts. Recent advances, including vascular-enrichment protocols for single-cell sequencing, spatial transcriptomics, and innovative human stem cell-based models, now offer unprecedented opportunities to dissect these intricate relationships with greater precision.

Encouragingly, the therapeutic landscape targeting the NGVU is expanding rapidly, encompassing multiple FDA-approved treatments and an emerging generation of cellular, molecular, and delivery-based approaches. Nonetheless, critical challenges remain in defining optimal intervention windows, refining patient stratification, and balancing therapeutic efficacy with the preservation of homeostatic NGVU functions. Moving forward, progress will depend on sustained interdisciplinary collaboration that bridges mechanistic discovery and clinical translation, ultimately advancing more effective interventions for NDD.

## Conflict of interest

All authors declare no conflicts of interest.
